# Current Perspectives on Clinical Use of Exosomes as a Personalized Contrast Media and Theranostics

**DOI:** 10.3390/cancers12113386

**Published:** 2020-11-16

**Authors:** Tomasz Lorenc, Julian Chrzanowski, Wioletta Olejarz

**Affiliations:** 1 Ist Department of Clinical Radiology, Medical University of Warsaw, 5 Chalubinskiego Street, 02-004 Warsaw, Poland; 2 Department of Biochemistry and Pharmacogenomics, Faculty of Pharmacy, Medical University of Warsaw, 02-097 Warsaw, Poland; s070007@student.wum.edu.pl (J.C.); wioletta.olejarz@wum.edu.pl (W.O.); 3 Centre for Preclinical Research, Medical University of Warsaw, 02-097 Warsaw, Poland

**Keywords:** cancer, diagnostic imaging, contrast media, personalized radiology, theranostics, exosomes, extracellular vesicles, MRI, CT, nuclear medicine

## Abstract

**Simple Summary:**

Precise and personalized radiology and nuclear medicine are in need of a new, biological contrast media and radionuclide. This inventive strategy is progressing due to the developing of contrast agents and radionuclides based on exosomes. The compatibility of exosomes with existing imaging modalities can accelerate incorporating these methods into clinical practice. Besides, a new generation of contrast media and radionuclides based on exosomes provides an opportunity to develop novel approaches in cancer diagnosis. Moreover, exosome-based diagnostic and therapeutic applications can open a new field in radiological departments called theranostics, combining simultaneous cancer diagnosis and therapy.

**Abstract:**

An appropriate combination of biomarkers and imaging technologies will become standard practice in the future. Because the incidence of and mortality from cancers is rising, the further study of new approaches for the early detection and precise characterization of tumors is essential. Extracellular vesicles (EVs), including exosomes, prove to have great potential when it comes to diagnosis and targeted therapy. Due to their natural ability to pass through biological barriers, depending on their origin, EVs can accumulate at defined sites, including tumors, preferentially. This manuscript discusses the difficulties and simplicities of processing cell-derived materials, packaging diverse groups of agents in EVs, and activating the biological complex. Developing exosome-based diagnostic techniques to detect disease precisely and early as well as treat disease marks a new era of personalized radiology and nuclear medicine. As circulating drug delivery vehicles for novel therapeutic modalities, EVs offer a new platform for cancer theranostic.

## 1. Introduction

In the area of emerging approaches in personalized medicine, the diagnostic accuracy of imaging is one of the main cornerstones of its success. EVs secreted by different cell types have great potential for personalized imaging as they favor accumulation in specific tissues, improve cargo distribution to specific sites in the body, and protect content from degradation [1]. When it comes to cancer diagnosis and therapy, some authors have proposed that tumor cells can capture their own EVs more efficiently in comparison with other cell-derived EVs. This suggests that tumor-specific proteins play essential roles in cellular uptake [2]. This property could facilitate the development of personalized radiology and nuclear medicine enhancing selectivity by targeting contrast media or therapeutic to tumor cells preferentially.

Exosome components can be engineered. When modified, they serve as nanocarriers, endogenously, at a cellular level, or exogenously, following cell culture production, collecting from body fluids [3]. The advantages of exosomes as nanocarriers outweigh many artificial nanovesicles [4]. Firstly, exosomes naturally carry RNA, lipid, protein, and metabolite cargo, thus enabling their use as contrast or drug delivery vehicles. Additionally, similar to cells, exosomes contain a deformable cytoskeleton and “gel-like” cytoplasm derived core. These biophysical properties enhance exosome structural integrity and resistance to rupture during trafficking in vivo [5]. EVs physiological and biochemical properties can be used as a therapeutic tool for drug delivery [6]. A number of reports demonstrating post isolation strategies to modify exosome surface structures have presented how exosomes in vivo are tracked more effectively [7]. 

Inherent structural, biocompatible properties, and post isolation modification of exosomes make them ideally suited as diagnostic carriers with great potential to serve in radiology and diagnostic imaging [8,9]. In this era, a theranostic approach using targeted exosomes has unique promise for personalized treatment of cancer, as both the targeting vehicle and the therapeutic can be tailored to the individual patient [5,10]. Individualized targeted theranostic nanomedicine has emerged as a promising solution to increasing sensitivity and specificity during diagnosis. By using nanoparticles in the diagnosis and therapy of patients, the likelihood of prolonged survival after therapy is far greater [11]. This review covers current and emerging strategies for imaging by means of exosomes in preclinical and clinical practice as well. We considered papers describing new diagnostic methods and the development of specific biological EVs to diagnose and treat human diseases, especially cancers. However, due to technical challenges as well as standardization in isolation, quantification, and characterization, their use as in vivo imaging in real clinical practice is hampered. As EVs are nanometer-sized, imaging them demands a full understanding of each labeling, loading, encapsulating, and modification strategy to ensure accurate monitoring. Here, these gaps will be highlighted and future opportunities in the study and reengineering of EVs for diagnostic imaging and cell-free therapy suggested. This review will cover the relevance of EVs both as a diagnostic and as a theranostic tool.

## 2. Structure and Function of Exosomes

EVs are classified into four major groups characterized by size and origin: exosomes, microvesicles, apoptotic bodies, and oncosomes [12]. Exosomes as the smallest EVs (diameter 30–100 nm) relay information between tissue microenvironments and can influence target cell function and differentiation [13]. They carry mRNA and miRNA, proteins, lipids, signaling molecules, and transfer into the target cells [14]. EVs are released by most cell types and circulate in all biological fluids, including blood, plasma, serum, saliva, and urine [15,16,17]. They fuse with the membrane of endosomes/lysosomes in an acidification-dependent manner causing cargo exposure to the cell cytosol [18]. Exosomes are created by endosome membrane invagination. This results in the creation of intraluminal vesicles within multivesicular bodies (MVBs) [19,20], formed through the release of intraluminal vesicles (ILVs) of multivesicular endosomes. It has been shown that exosomal biogenesis is implicated in the components of the Endosomal Sorting Complex Required for Transport (ESCRT) [21]. 

**Figure 1:**
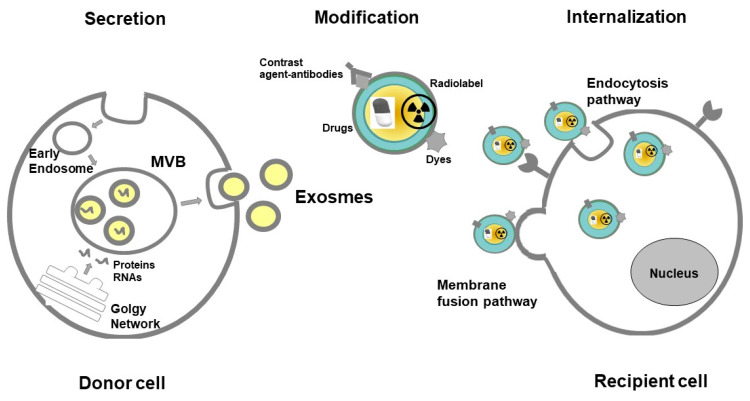
Pathway of exosome secretion and internalization in the human body for diagnostic and therapeutic purposes. Abbreviations: MVB—Multivesicular body.

EVs contribute both to physiology as well as to pathology and protect cells from accumulation of waste [22]. They play a key role in physiological balance and homeostasis and participate in intercellular signaling and communication [23,24]. EVs are responsible for a large variety of biological processes, such as immune surveillance, and regulation of inflammation [25]. EVs can deliver pathogenic agents to non-infected cells and they are thought to condition the tumor microenvironment [26]. It was shown, that tumor-derived exosomes (TEX) are responsible for cancer development, metastasis, and progression by suppression of immune cells [27]. They are involved in angiogenesis in cancer progression by transporting pro-angiogenic biomolecules [28]. Exosomes have potential clinical applications as biomarkers and drug carriers in anticancer therapy [29,30] and provide strong potential for imaging of cancer cells [9]. Figure 1 depicts a possible pathway of exosome secretion and internalization in the human body for diagnostic and therapeutic purposes.

## 3. Isolation and Analysis of Exosomes

The International Society for Extracellular Vesicles (ISEV) does not clearly define the issue of extracellular vesicles (EV), leaving their isolation to the discretion of investigators [12,31]. Until now, ultracentrifugation (UC) is the most commonly applied EV primary isolation method [32]. These methods include: density-gradient centrifugation (DGC) [33], sucrose cushion centrifugation [34], size exclusion chromatography (SEC) [35], affinity chromatography (AC) [36,37], membrane filtration [38] as well as the most recently established AF4 technique [39,40]. Despite this method being commonly applied, it does have several drawbacks. These include low reproducibility, a low RNA yield, low sample throughput, and potential exosome damage, incompatible with clinical utilization [41]. As it turned out the second often used method for isolation of exosomes utilizes SEC for sourcing of exosomes from cell-line supernatants or cancer patients’ plasma. In the case of this method, isolated exosomes are functionally competent and their molecular content parallels that of the parent tumor cells [42]. 

EVs physical features, like morphology, size, distribution, and concentration, are measured with microscopic methods [43]. The most common techniques used in EV studies are flow cytometry (FC), nanoparticle tracking analysis (NTA), dynamic light scattering (DLS), scanning and transmission electron microscopy (SEM and TEM) as well as atomic force microscopy (AFM) [44,45]. Electron microscopy (EM) and atomic force microscopy (AFM) present high resolution, producing EV images [46]. SEM and TEM present EV sample images by means of surface scanning with a focused beam of electrons. In this way, three-dimensional surface topography information is gathered together with a sample’s elemental composition [47]. AFM is a high-resolution imaging technique applied for EV characterization. It works with the help of amplitude modulation, which detects changes in the amplitude of cantilever vibration to collect information regarding surface topography. Data regarding local stiffness and adhesion properties are gathered by using phase modulation, as it records energy dissipation [48]. DLS is used for measuring bulk scattered light from EVs. This takes place with illumination facilitated by a monochromatic light source [49]. NTA determines particle concentration and size distribution, thus it is a particle tracking method [50]. DLS and NTA apply the Brownian motion theory. This happens when analyzing random changes of light intensity scattered by particles in solution. They differ, though, as DLS measures bulk scattering while NTA tracks individual particle scattering. Upon application, concentration is calculated (i.e., the number of particles in the view field) and size distribution (i.e., hydrodynamic diameter through the Strokes–Einstein equation) [51,52]. The method for quantitatively evaluating the amount of exogenously administered exosomes delivered to each organ is ^125^I-labeling of exosomes using the SAV-biotin system. Radioisotope ^131^I-labeled exosomes from diverse cellular origins, e.g., endothelial progenitor cells, myeloid-derived suppressor cells, and tumor cells can be utilized to monitor disease progression, metastasis, and targeted therapy [53]. The major organ in the clearance of exogenously administered cell-derived exosomes is the liver [54]. 

## 4. Modification of Exosomes for Imaging and Theranostic Application

### 4.1. Re-Engineering Strategies

As exosomes are small in size, disperse rapidly in body fluids, and have a similar to body cell composition resulting in a lack of contrast for imaging techniques, tracking them in live organisms is somewhat demanding [55]. Therefore, two main approaches have been developed [7]. These are direct and indirect labeling pathways [56]. The former is based on loading exosomes with labeling agents upon isolation. The latter, on the other hand, involves manipulating parent cells by introducing exogenous agents. They are then later incorporated into secreted exosomes. Both pathways are suitable and reliable for real-time exosome in vivo imaging [57]. 

### 4.2. Encapsulating and Loading Exosome with Contrast Agents

The exosome lipid bilayer membrane is a natural barrier protecting exosome load from degradation in the circulatory system. However, this membrane, just as endogenous exosome content, makes exosome loading with contrast agents or drugs exceptionally challenging [55]. Hydrophobic insertion, covalent surface chemistry, and membrane permeabilization are strategies used for direct EV loading and labeling [58]. Most of the strategies involve exosome labeling outside the body. They are then delivered back to the body. Distinctive imaging methods and modalities have been applied to visualize the marked exosomes. Each strategy has various capabilities and advantages [7]. The exogenous labeling procedure utilizes an external agent that locates nanoparticles or dye within the exosome. This kind of labeling is simpler to apply. Furthermore, it permits the utilization of additional in vivo imaging modalities like MRI and CT. However, these labeling agents can conceivably diffuse out of the exosomes (e.g., when the exosome raptures). As a result, careful outcome confirmation and interpretation are required [59].

### 4.3. Loading the Parent Cell before Exosomes Release

**Figure 2:**
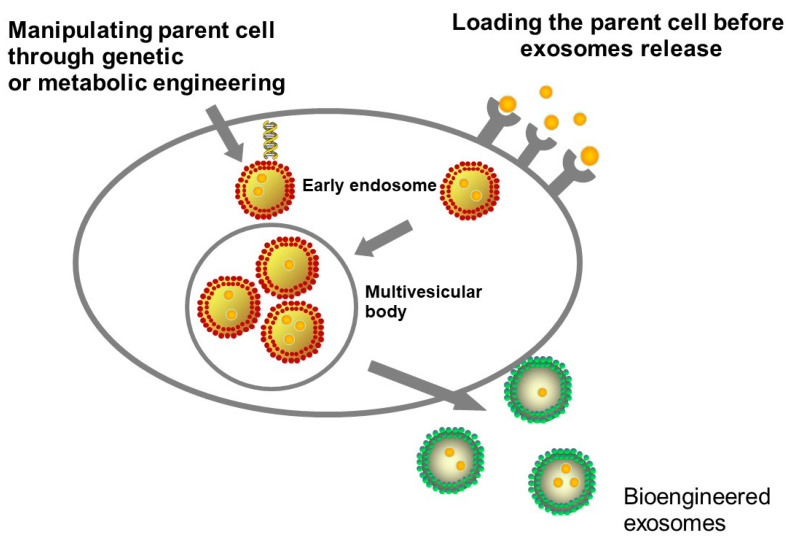
Loading strategies may rely on pre-loading parental cells with the cargo of interest before inducing extracellular vesicles (EVs) release or manipulating parent cell through genetic or metabolic engineering.

EVs can be adjusted by manipulating parent cells through genetic or metabolic engineering, or by introducing exogenous material subsequently incorporated into secreted EVs [58]. Modifying parent cells is generally accomplished by seizing biosynthesis to support the creation of specific endogenous material or by delivering exogenous species to the cytoplasmic membrane [58]. The two methodologies can be utilized to manipulate cells to secrete modified EVs. The latter approach may additionally be used to directly functionalize purified EVs [58]. Engineering parent cells to functionalize and enclose molecules or nanoparticles in EVs, for imaging or therapy purposes are shown in Figure 2.

### 4.4. Adapting Exosome Surface Structures and Functionalization

**Figure 3:**
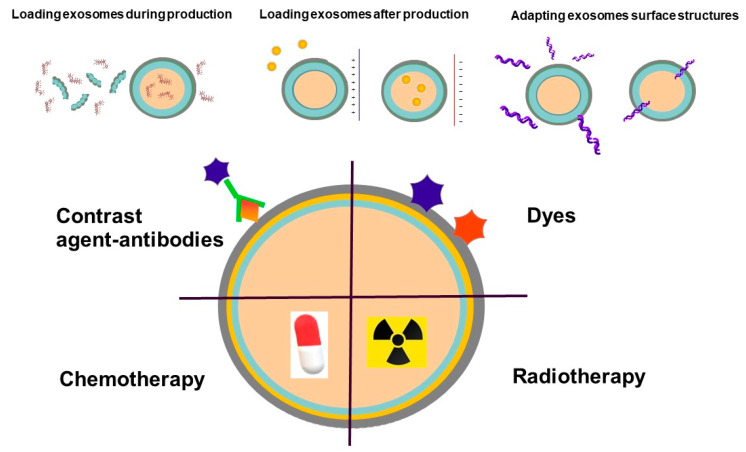
Strategies for EVs modification. A direct EVs modification strategy is to permeabilize the vesicle membrane to allow the active loading of molecules into the EVs interior, an approach that has been exploited for drug delivery. Agents can be incorporated into EVs, for instance, as contrast media or radionuclide.

Engineered EVs are equipped with incorporated stimuli-responsive elements as well as targeting ligands and are characterized by immune evasion properties [60]. Altering an EV’s surface can have an impact on targeting capabilities and biodistribution. Transport properties, on the other hand, can be altered by removing endogenous surface molecules [61]. EV functionality can be introduced by adding peptides sensitive to the environment, e.g., EV-based drug delivery by utilizing pH-sensitive functional groups (tumors have an acidic extracellular microenvironment [pH 6.5–7.2], while all cells have an acidic intracellular endosomal environment [pH 5.0–6.5] compared to the physiological pH of 7.4). Various strategies have been developed to stop the immune system from being activated. These include polymer coatings that decrease interactions with cells as well as pre-treatment strategies that deactivate macrophages. Surface modification with polyethylene glycol (PEG) is the most common approach among these strategies [60]. Antigen-specific antibody light chains could coat the surface of EVs to increase cell targeting specificity, while EV membrane lipids may possess immune adjuvant activity [62]. Engineering EVs to functionalize and enclose molecules or nanoparticles, for imaging or therapy purposes are shown in Figure 3.

A comparison of the pros and cons of different exosome modification techniques is included in Table 1.

## 5. Different Radiological Modalities and Nuclear Imaging Which Can Utilize Exosomes as a Contrast Agent

### 5.1. Computed Tomography 

The size of exosomes makes it impossible to directly use computed tomography as a means for visualization. To do so, one needs to include some form of a contrast agent. Gold nanoparticles are diagnostic agents displaying physical properties facilitating analysis with the use of high-resolution imaging techniques, including CT. This enables quantitative analysis deep inside the body, and also possesses the additional advantage of being highly biocompatible [63,64]. Lara and colleagues used folic acid-conjugated gold nanoparticles. This way, cell internalization is promoted and trafficked through the endocytic/MVB pathway for subsequent secretion within EVs [65]. The natural tropism of EVs was not altered by this methodology. As a result, it can be applied to study EV distribution in terms of diagnostics. When comparing 4 different EV types, it was observed that melanoma cells take up their own EVs preferentially [65]. This study focuses on EV melanoma tropism towards cancer cells and metastatic tumors. It also determines its potential for drug delivery strategies and novel contrast agent composition.

Gold was also used as a contrast agent in a study conducted by Perets and colleagues [66]. They have acquired exosomes from mesenchymal stem cells (MSC) and labeled them with glucose-coated gold nanoparticles (GNP). The proposed mechanism of action by which GNPs uptake into MSC-derived exosomes took place through an active energy-dependent system mediated by the glucose transporter GLUT-1, involving endocytic proteins. It was also presented that intranasal administration leads to a better brain accumulation of MSC exosomes in comparison to the intravenous route. In vivo CT imaging showed that in a stroke model C57bl/6 mouse, where focal ischemic damage was induced with endothelin-1 intrastriatal injection, intranasally administered labeled MSC exosomes presented homing specifically to injured brain regions [67]. In a follow-up study, the scope of tested neuropathologies was broadened by autism, Parkinson’s disease, and Alzheimer’s disease. The results confirmed previous research outcomes. MSC derived GNP labeled exosomes presented good migration and homing properties toward specific neuropathology areas, specifically to neurons. Moreover, it was concluded that the innate immune response associated with inflammation and ligands associated with chemotaxis plays a role in these homing processes [66]. Studies on EVs have already been described and are summarized in Table 2. 

### 5.2. Magnetic Resonance Imaging (MRI)

MRI is a remarkable imaging technique, which offers anatomic information with high and versatile soft-tissue contrast. Gadolinium-based components are most commonly used for contrast enhancement. Moreover, two types of iron oxide contrast agents exist: superparamagnetic iron oxide (SPIO) and ultrasmall superparamagnetic iron oxide (USPIO). Most published articles have addressed the idea of loading magnetic nanoparticles (NPs) within exosomes. This provides them with multiple capabilities as contrast agents in MRI while simultaneously serving a role as a drug vehicle [68,69]. This is when EVs are most commonly labeled with superparamagnetic iron oxide nanoparticles, which are sized between 5 and 150 nm [69,70,71]. Unfortunately, due to typically low tissue concentration of EVs, there is a lack of MRI technology with sufficient sensitivity (normally a 10 μM to 10 mM contrast agent is needed for MRI; however, the picomolar concentration of a radiotracer was enough for positron emission tomography **(**PET) or single-photon emission computed tomography (SPECT) imaging) [71,72]. Hood et al., optimized the electroporation procedure to load 5 nm superparamagnetic iron oxide nanoparticles (SPIONs) in exosomes while minimizing their aggregation [68]. A new approach for USPIO exosome labeling was reported by Busato and colleagues. It enables MRI detection while preserving morphological and physiological characteristics [70,73]. At first, mesenchymal stem cells were labeled using USPIO. Next, exosomes were isolated using a standard isolation protocol. Exosomes isolated from previously labeled cells retain nanoparticles. Exosome labeling efficiency was assessed in in vitro and in vivo animal model by MR imaging acquisition. This was also carried out by determining the magnetic resonance (MR) image contrast. Having efficiently visualized exosomes-USPIO in vitro, the focus was placed on the in vivo MRI detection in mice intramuscularly injected with labeled exosomes. Exosomes-USPIO were clearly detectable in in vivo MR images in the muscular tissue. It was inferred that USPIO are an optimal candidate for exosome labeling; the reason being that they are stable and biocompatible, ranging in size from 5 nm to 7 nm. Therefore, they are small enough to be incorporated in exosomes [70,73]. 

In recent studies, the electroporation technique has been used to label exosomes with USPIO [69]. However, electroporation may affect membrane integrity. When the membrane is exposed to a strong electric field, it causes spontaneous pore formation. Nevertheless, a method enabling exosomes to be loaded with superparamagnetic iron oxide nanoparticles via electroporation was reported by Hu and colleagues. It allows imaging of in vivo loaded exosomes within lymph nodes (LN) in a mouse model with melanoma [69]. 

Using MRI imaging Zhu et al. have performed an evaluation of an antigen, which is a prostate-specific membrane antigen (PSMA) that targets SPIONs. The specific uptake of polypeptide-based SPIONs with PSMA expressing cells were observed. It turned out that the MRI signal could specifically be enhanced. PSMA-targeting SPIONs may provide a new approach for prostate cancer (PCa) imaging [74]. 

Superparamagnetic iron oxide nanoparticles conjugated with rhodamine were used by Dąbrowska and colleagues for the labeling of vesicles in human bone marrow mesenchymal stem cells [75]. It has been shown that labeling dramatically alters the T2 relaxation time of EV suspension in vitro. This could turn out to be promising for future detailed studies on in vivo EV detection [75]. Liu et al. designed a new label method that allows the visualization of labeled exosomes from mesenchymal stem cells in vivo by MRI [76]. Ferritin heavy chain (FTH1) was used as an MRI reporter and replaced with the outer membrane part of lactadherin (a protein mostly located on the outer surface of exosomes). MRI images clearly showed a pronounced signal intensity contrast of labeled exosomes in mice. This, therefore, confirmed in vivo MRI exosome detection from mesenchymal stem cells [76].

Rayamajhi and colleagues explored EV reconstruction with gadolinium and liposomes. This enabled the development of a biomimetic contrast agent for contrast-enhanced MRI [77]. Gadolinium infused hybrid EVs showed specificity to cancer cells both in vitro and in vivo, so their application in cancer diagnosis and treatment monitoring is being explored. This could potentially enable proficient diagnosis with a minimal dose, as maintaining contrast in the clinical window was achieved by a three-fold reduction of clinically used Gd concentration. Moreover, gadolinium infused hybrid EVs show excellent contrast enhancement and enhanced retention ability in blood vasculature with no detected extravasation to interstitial spaces and tissues [77]. 

Reduced Gd dosage, prolonged observation in the vasculature, no extravasation into surrounding tissue during dynamic imaging may increase the safety of gadolinium application, especially in the evidence of body and brain (mainly dentate nucleus and globus pallidus) gadolinium deposition after serial injection of nowadays commercially available gadolinium-based contrast agents [78].

More supplementary information about the potential role of EVs in MR imaging is also provided in Table 2.

### 5.3. Ultrasound (US)

Osborn and colleagues demonstrated the ability of the reconstituted exosomes to generate both linear and nonlinear response to ultrasound excitation and act as ultrasound contrast agents [79]. The authors made bovine milk-derived exosomes echogenic by freeze-drying them in the presence of mannitol and assessed the echogenicity of the prepared exosomes. Researchers registered more than a three-fold increase in the brightness of the kidney when the studied exosomes were injected into the vein [79]. 

An emerging technique for drug delivery localization is the use of the ultrasound in conjunction with gaseous microbubbles (MB). Sun and colleagues used ultrasound to transport exosomes into tissues that are reluctant to them: heart, adipose tissue, and skeletal muscle. This technique, called ultrasound-targeted microbubble destruction (UTMD), was described [80,81] and is based on low-pressure and high-pressure ultrasound waves. In the low-pressure setting, the microbubbles (MB’s) expand and contract inversely, proportional to acoustic pressure waves—a process called stable cavitation. However, if the acoustic pressure is high enough, MB’s cavitate non-linearly, which leads to implosion and collapsing—a process called inertial cavitation. Both cavitation mechanisms have the same effect: breakdown of cell junctions, cell membrane perforation, and tissue permeabilization. They have injected DiR/Dil fluorescent dye-labeled exosomes into living C56BL/6 mice and compared it with mice that were given a combination of dye-labeled exosomes and SonoVue^TM^ microbubbles. The results of this comparison revealed that the targeted destruction of the microbubbles in the aimed region significantly facilitates the exosome endocytosis and thus enables exosome transport into reluctant tissues. Interestingly, increasing the ultrasound exposition time from 0.5 min to 3 min did not increase the exosome’s infiltration linearly, as expected by the author [80,81]. 

In a study conducted by Li and colleagues, an analogous method to UTMD was used. This approach exploited focused ultrasound waves (FUS) affecting microbubbles to disrupt blood–brain barrier, which enables exosome penetration. Two types of exosomes were used: blood serum-derived and macrophage-derived. All of them were labeled with DiR fluorescent dye with part of exosomes being loaded with doxorubicin and injected into C57BL/6 mice bearing orthotopic glioma (GL261) tumors. The results showed that both exosome types could effectively deliver a chosen drug. Here, doxorubicin was delivered into glioma tumors with the help of FUS, while no significant differences in physical features, ultrasound-responsiveness, or specific glioma targeting were observed [82]. Moreover, another modality of ultrasonography called low-intensity pulse ultrasonography (LIPUS) as well as its impact on exosomes was studied by Li and colleagues [83]. Bone marrow dendritic cells (BMDC) were extracted from dead C57BL/6 mice’s bone marrow and part of them was treated with LIPUS. Next, exosomes were extracted from both BMDC LIPUS treated and untreated cells, labeled with DiD, and incubated with human umbilical vein endothelial cells (HUVECs). A comparison between HUVECs incubated with LIPUS treated BMDC exosomes and HUVECs incubated with LIPUS untreated BMDC exosomes was made by adding tumor necrosis factor α (TNF-α). The result was a highly stimulated expression of intercellular adhesion molecule 1 (ICAM-1) and vascular cell adhesion molecule-1 (VCAM-1) in the LIPUS untreated group and markedly reduced induction of ICAM-1 and VCAM-1 in LIPUS treated group. It was also shown that LIPUS treated exosomes exhibited increased amounts of miR-16 and miR-21 which are responsible for blunting nuclear factor kappa-light-chain-enhancer of activated B cells (NF-κB) signaling pathway and thus mitigate TNFα-elicited endothelial inflammation [83].

Besides, ultrasonography presents unique properties that can be exploited in extracellular vesicle research. The ultrasound, in combination with microbubbles, was capable of loading drugs in endothelial cells and simultaneously triggering the release of EV-carrying drugs. This highlights the potential of EVs as drug nanocarriers for future drug delivery in cancer [84]. A team of scientists led by Zhao used a low-intensity ultrasound (LIUS) on the A2780 ovary cancer cell model. An intensity of 0.5 W/cm^2^ for 60 minutes resulted in a significant increase in exosome production by these cells. As stated by the author, the resultant exosomes had no notable change in morphology, size, or in vivo distribution. The suspected mechanism of action by which ultrasound waves can increase exosome production is its influence on parts of EV’s cell metabolism such as ESCRT complex, Rab GTP-ases, and *TSAP6* gene. An additional advantage to using LIUS is its high penetrance, meaning it could be used to irradiate large cell cultures, i.e., in a bioreactor [85]. 

### 5.4. Nuclear Imaging—Single Photon Emission Computed Tomography (SPECT) and Positron Emission Tomography (PET)

Emission based imaging could be another modality for utilizing exosomes. A method for the radiolabeling of macrophage-derived exosome-mimetic nanovesicles (ENVs) with ^99^mTc-HMPAO under physiologic conditions was demonstrated in research conducted by Hwang and colleagues [86]. Macrophage cell line derived from RAW264.7 mice was used to produce ENVs. These nanovesicles were then labeled during incubation. The distribution of this intravenously administered technetium derivative was monitored in vivo using SPECT in living BALB/c mice. Nuclear imaging showed a difference in the distribution of radioisotope depending on whether it was injected alone or in an ENV. The brain did not show any uptake of ENV encapsulated technetium, in opposite to technetium alone. Nevertheless, both presented high signals in the liver. It was also stated that the expression of exosome specific protein (CD63) did not change in ^99^mTc-HMPAO-ENVs as well as that radiochemical purity of those vesicles was higher than 90% [86]. A different source of EVs was shown in a study led by Varga [87]. These erythrocyte-derived EVs were labeled using ^99^mTc-tricarbonyl complex. After injecting the aforementioned EVs into BALB/c mice, a series of images were produced using SPECT. The technetium labeled exosomes showed a high level of accumulation in the liver and spleen. According to the author, only a minor fraction of the radioactive label became detached from the EVs [87]. 

Furthermore, Smyth et al. decided to acquire exosomes from in vivo unmodified tumors and compare them to PC:Chol liposomes and liposomes made from the lipid extract of exosomes using reconstituted ^111^In-oxine as a radiolabel [88]. Through intravenous administration, all three tested substances showed comparable biodistribution profile and rate of clearance. Nevertheless, unmodified tumor-derived exosomes, when given intratumorally, remained associated with tumor tissue to a greater extent than PC:Chol liposomes. Moreover, this study showed that the innate immune system along with the complement protein C5 has a significant impact on exosomes’ rate of clearance [88]. 

Another study used a unique approach to labeling exosomes using radioisotope ^99^mTc (IV) in an ionic salt form (^99^mTcCl_4_) [89]. Using this technetium salt on natural milk-derived exosomes enabled longitudinal tracking by SPECT imaging. In comparison to previously described radiolabeling methods using ^99^mTc-HMPAO or ^99^mTc-tricarbonyl, this approach is less expensive and complex as well as less impactful on the structure of exosomes, according to the author. This method also demonstrated the pivotal role of the administration route in the biodistribution of the exosomes [89].

Royo et al. labeled EVs directly with [^124^I]Na. They were then injected in mice via the intravenous route or into the hock [90]. Using PET, the amount of radioactivity in major organs was measured at different time points after administration. It was found that intravenous injection leads to quick EV accumulation in the liver. The other group of EVs taken from mouse liver proliferative cells was treated with neuraminidase. This enzyme digests terminal sialic acid residues from glycoproteins. Comparing with intact EVs, glycosidase treatment induced accumulation in the lungs. It is worth noting that altering glycosylated complexes on the EV surface, it has an impact on vesicle distribution. Furthermore, when removing sialic acid residues, more EVs reach and accumulate at the lungs [90]. Various modalities, such as nuclear, CT, and MRI used for EVs imaging have been summarized in Table 2.

### 5.5. Hybrid Imaging 

The abnormal state at the target site of the related disease may be visualized with the powerful tool of multimodal bioimaging. In their study, Shaikh and colleagues used multimodal imaging techniques to boost earlier and more accurate tumor diagnosis. These included computed tomography, fluorescence, and magnetic resonance imaging [95]. The facile in the situ biosynthesis of iridium and iron oxide nanoclusters (NCs) in cancer cells or the tumor tissue was reported. Shaikh et al. demonstrated that highly magnetic NCs are both biocompatible and tumor-targeted; NC formation fails to take place in normal cells or tissues. Iridium(III) chloride hydrate and FeCl_2_ pre-ionic solutions that converted to IrO_2_ and Fe_3_O_4_ NCs within the neoplastic cells in vitro and xenograft murine models in vivo were used as probes for CT and MRI multimodal cancer bioimaging. Ir−Fe ion treatment for CT imaging was evaluated, and it turned out that biosynthesized NCs successfully served as CT contrast agents and probes for bioimaging. What is more, it was observed that 24 h upon Ir-Fe injection, the tumor region significantly darkened on T2-weighted MRI. A relatively high signal intensity, as compared to the control, was noted. In addition, exosomes were isolated and the biosynthesized NCs internalized within exosomes. Such exosomes can be used as cancer biomarkers. Exosomes were also isolated from xenograft mice serum. Biosynthesized IrO_2_ and Fe_3_O_4_ NCs are internalized within exosomes, and so they could subsequently be used as potential biomarkers for cancer diagnosis and imaging [95]. 

A similar study was performed by Tayyaba and colleagues [96]. In this study, tumorous cells were utilized for the in situ biosynthesis of silver and NCs from respective salts. The self-assembled biosynthesized silver and iron nanoclusters were readily loaded into exosomes as payloads. They were then secreted into environments. It is believed that Fe_3_O_4_ NCs loaded exosomes have potential as a contrast agent both for CTs and MRIs [96]. 

Banerjee et al. reported a two-step surface modification methodology. In it, small extracellular vesicles (SEVs) with ^64^CuCl_2_ were radiolabeled for PET/MRI imaging [71]. Following intravenous administration, the biodistribution of radiolabeled SEVs could be monitored by PET and by MRI. Modification failed to alter morphology, surface receptor proteins or internal RNA content of small extracellular vesicles. Thus, the authors concluded that their labelling strategy may be useful for diagnostics and therapies based in SEVs as it is relatively simple and very sensitive. The main studies on multimodal imaging are summarized in Table 3.

## 6. Exosomes as Theranostic 

**Figure 4:**
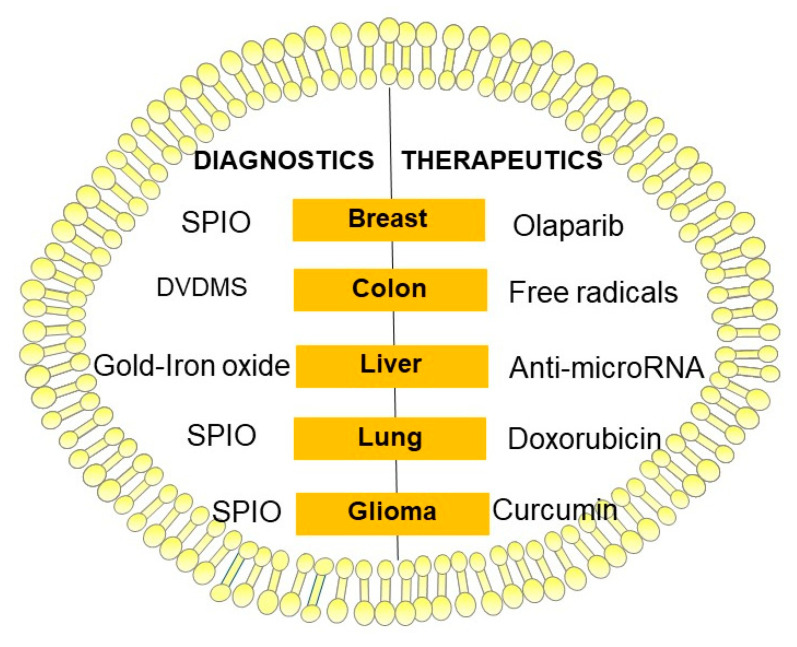
Schematic diagram illustrating exosomes as theranostic. Abr. DVDMS—sinoporphyrin sodium; SPIO—superparamagnetic iron oxide.

Finding a way to utilize exosomes as a contrast agent and a cargo for therapeutic molecules at the same time is a challenge (Figure 4). Bose and colleagues investigated tumor cell-derived extracellular vesicles for multimodal miRNA delivery and phototherapy treatments as well as cancer MRI [97]. They demonstrated anti-miR-21 loading that blocks the function of endogenous oncogenic miR-21 and exosome coating with Gold–Iron Oxide Nanoparticles. The iron oxide acts as a strong T2 contrast agent for MRI, and the gold coating on the iron oxide core acts as a photosensitizer by converting deeply penetrating near-infrared (NIR) light to heat (wide absorption spectrum of gold). T2-weighted gradient-echo imaging showed a significant accumulation of labeled exosomes in tumors. The MRI signal change showed a strong T2 signal reduction in animals receiving labeled exosomes in comparison with control mice [97]. Gold-based NPs are a workhorse in nanomedicine, with applications in widely different fields including imaging [98]. Recent work aimed at combining the therapeutic capabilities of hollow gold nanoparticles (HGNs) with exosome unique tumor-targeting properties [99]. The authors developed highly efficient methods of the plasmonic nanoparticles encapsulation into exosomes used in photothermal ablation [99]. 

Combined loading of drugs and nanoparticles in EVs can be done following incubation with various nanoparticles to allow internalization [100]. In the study carried out by Silva and colleagues, vesicles derived from macrophage (THP-1) cells were successfully loaded with iron oxide nanoparticles (ION) and several therapeutic molecules irrespective of their molecular weight or hydrophobicity [101]. Different magnetic vesicles loaded with either a chemotherapeutic drug (doxorubicin), an anticoagulant protein (tissue-plasminogen activator (t-PA)), or two photosensitizers were produced [101]. However, these techniques are of low yield, thus limiting its application.

Jia and colleagues loaded SPIONs and curcumin (Cur) into exosomes. They then conjugated the exosome membrane with neuropilin-1-targeted peptide to obtain glioma-targeting exosomes with imaging and therapeutic functions [102]. When administered to glioma cells and orthotopic glioma models, it was found that the engineered exosomes could cross the blood-brain barrier smoothly, thus providing good results for glioma targeted imaging and therapy. Furthermore, SPION-mediated magnetic flow hyperthermia and curcumin-mediated therapy also showed a potent synergistic antitumor effect [102]. Srivastava et al. have demonstrated an application of exosomes as theranostics in lung cancer [103]. They used normal lung fibroblast cell-derived exosomes and attached doxorubicin (Dox), an anticancer drug, and 5–10 nm SPION. Authors demonstrated that they can deliver anticancer therapeutics (Dox) to lung cancer cells and simultaneously image them using MRI [103].

Jung and colleagues had chosen hypoxic human breast cancer-derived exosomes to be carriers for Olaparib and SPIO nanoparticles for magnetic particle imaging (MPI). Diagnostic value and therapeutic efficacy of hypoxic exosomes were tested in vivo, on mice bearing breast cancer xenografts. MPI images showed that cells and exosomes transfected with SPIO have a strong MPI signal and this signal increased linearly as a function of exosome number. In vivo testing confirmed therapeutic results, showing a significant delay in the growth of tumors treated with Olaparib-loaded hypoxic exosomes. However, in in vivo model, the signal from SPIO-loaded hypoxic exosomes in MPI/CT imaging was primarily observed in the liver and did not yield a detectable MPI signal in the tumor [104].

In research conducted by Liu and colleagues, a sonodynamic therapy (SDT) was used as a means for theranostics [105]. In their study, a functionalized smart nanosonosensitizer (EXO-DVDMS) was made. This was done by incorporating sinoporphyrin sodium (DVDMS), an ultrasound responsive agent, into mouse 4T1 mammary cancer cell-derived exosomes. As SDT relies on the synergistic effects of ultrasound and the sonosensitizer, free radicals i.e., singlet oxygen derived from the sonosensitizers following ultrasound stimulation are highly cytotoxic and contribute to this method’s anti-cancer results. What is worth noting is the fact that EXO-DVDMS showed a significantly higher singlet oxygen yield than free-DVDMS. This signifies that exosomal coating improves the stability of free DVDMS. The cancer cell uptake and targeted delivery of EXO-DVDMS were assessed through exosome labeling with DiO dye. It resulted in EXO-DVDMS exhibiting intrinsically superior selectivity to homologous tumor cells (4T1) in vitro than other tested cells. Exposing EXO-DVDMS to ultrasound waves increased the oxidative stress even more. This is the probable mechanism of SDT’s anti-cancer effect. The findings of in vivo testing on 4T1 tumor-bearing (homotypic tumor) and CT26 tumor-bearing (non-homotypic tumor) mouse models confirmed SDT anti-cancer properties. Additionally, it was shown that ultrasound waves caused a subcellular location shift of EXO-DVDMS from lysosomes to the mitochondria, indicating SDT-induced lysosome degradation and endosome opening, thus initiating cell death-signaling pathways. This exosomal formulation was also proved to facilitate simultaneous imaging and tumor metastasis inhibition [105]. Table 4 summarizes the current main methods detailed in this review. (Table 4).

## 7. Potential Role of the Exosomes in Cancer Imaging and Theranostic Application

### 7.1. Breast Cancer

Breast cancer is the most widespread form of female cancer. CA 15-3 being the most applied serum marker in metastatic breast cancer patients [106,107]. A molecular profile of exosome miRNAs secreted from breast cancer cells showed that there was a higher level of miR-1246 and miR-21 in breast cancer patient plasma exosomes. Findings indicate that the plasma exosome miR-1246 and miR-21 combination formed a better indicator of breast cancer compared with individual marker measurements [108]. 

DiO, a fluorescent lipophilic tracer, was used to label exosomes isolated from MDA-MB-231 human breast cancer cells to quantify their uptake by hypoxic cancer cells and was modified to carry SPIONs and Olaparib (PARP inhibitor). Subsequently, the exosomes were monitored in vivo using MPI. Increased apoptosis and slower tumor growth in vivo confirmed the therapeutic efficacy of Olaparib-loaded exosomes [104]. 

Loading silicon quantum dots (30 nm) and gold-carbon quantum dots onto the exosome’s outer membrane (50−100 nm) caused a high-resolution image of live cells and the metastatic activity of breast tumor cells with minimal cytotoxicity [109,110]. Exosomes modified with fluorescence radiolabeled and PEG could image a higher resolution from 4T1 breast cancer cells and provide a visual examination of cellular uptake in a mice model [92]. 

Exosomes cross-linked with alkyne groups using carbodiimide chemistry conjugated to azide-fluor 545 may be used for fluorescent imaging [111]. 

### 7.2. Prostate Cancer

Prostate cancer (PCa) is the most common solid malignant cancer worldwide while the prostate-specific antigen (PSA) is the most widely used blood-borne biomarker for screening prostate cancer [112]. Exosomes may by key biomarkers for the diagnosis of prostate cancer [30]. They can be detected and isolated from various body fluids for prostate cancer diagnosis [113]. Exosomal miR-34a induce docetaxel sensitivity in docetaxel resistant prostate cancer cells by inhibiting Bcl-2. Exosomal miR-34a can be used as a predictive biomarker for its response to docetaxel [53]. Additionally miR-182 of the miR-183 cluster family was detected in prostate cancer cell derived exosomes from the serum [61]. Confocal imaging indicated, that exosomal miR-141-3p from MDA PCa 2b promoted osteoblast activity and increased osteoprotegerin OPG expression. In mice injected with miR-141-3p-mimics exosomes observed apparent osteoblastic bone metastasis [114].

Gold nanoparitcles of 13 nm diameter conjugated with anti-miRNA21 were incorporated in human prostate PC-3 cell derived exosomes by Alhasan et al. [115]. Altanerova et al. reported that iron oxide labeled mesenchymal stromal cell exosomes accumulate within prostate cancer cells in vitro. Such cancer cells can be killed by magnetic hyperthermia [116].

### 7.3. Lung Cancer 

Lung cancer (LC) is the main cause of cancer-related death worldwide [117]. The current standard diagnostic procedures typically involve imaging methods like CT and invasive transbronchial needle aspiration or transthoracic biopsy [118]. Examination of exosomes from plasma of 276 non-small cell lung cancer (NSCLC) patients shows, that CD151, CD171, and tetraspanin 8 are the strongest separators of patients with LC of all histological subtypes [119]. It was shown, that also miRNAs like miR-33a-5p/miR-128-3p in whole blood may serve as novel biomarkers for the early detection of LC. MiR-33a-5p and miR-128-3p in LC tissues were significantly correlated to cancer stages. MiR-128-3p in LC tissues were also remarkably related to tumor size [120]. 

The use of ION (5−10 nm) and doxorubicin in exosomes derived from lung fibroblast cells were able to capture a high-resolution tumor in addition to its treatment activity in lung cancer cells (H1299 and A549) [103].

A study by Royo and colleagues showed that the modification of glycosylated complexes on the EV surface can affect their distribution. By removing sialic acid residues, more EVs reach and accumulate at the lungs [90]. The authors labeled EVs directly with [^124^I]Na and the amount of radioactivity in major organs was measured at different time points after in vivo administration using positron emission tomography [90]. 

### 7.4. Hepatocellular Carcinoma 

Hepatocellular carcinoma (HCC) is the main cancer worldwide and remains one of the most common causes of cancer-related death globally [121]. Exosomal miRNAs may have potential value in the early diagnosis and treatment of HCC. Increase miR-21, miR-221, and miR-222, and decreased miR-122-a, miR-145, miR-199-a, and miR-223 are associated with the occurrence and development of liver cancer [122].

Tayyaba and colleagues used HepG2 liver cancer cells for the in situ biosynthesis of silver and iron oxide nanoclusters [96]. Biosynthesized by parent cells, silver and iron nanoclusters were readily loaded on the exosome as payloads. They were then secreted into the cell culture medium. Fe_3_O_4_ loaded exosomes have potential as a contrast agent for CTs and MRIs [96].

Bose and colleagues produced Cy5-anti-miR-21-loaded TEVs from HepG2 liver cancer cells and confirmed their functionalization by gold-iron oxide nanoparticles, which may act as a magnetic resonance imaging of cancer as well as phototherapy treatments [97]. 

### 7.5. Glioblastoma

Glioblastoma multiforme (GBM) is the most commonplace primary central nervous system malignancy [123]. GBM secretes large quantities of cancer-specific EVs. They may pass out through the blood–brain–barrier into the circulation. GBM EVs contain both mRNA, miRNA, and angiogenic proteins. Thus, they can stimulate tubule formation in endothelial cells and deliver functional RNA to recipient cells [124].

Jia and colleagues reported on the construction of targeted exosomes synchronously loaded with superparamagnetic iron oxide nanoparticles and curcumin by electroporation and then conjugated with neuropilin-1-targeted peptide [102]. It was demonstrated that these exosomes have a strong glioma-targeting ability, which could help in the early glioma diagnosis and the evaluation of the curative efficacy of drugs. Furthermore, the therapeutic effect exerted by glioma-targeting exosomes finally led to a survival benefit. This was very valuable and difficult to accomplish [102].

Bai and colleagues demonstrated that the combination of focused ultrasound and naturally abundant blood serum-derived exosomes are a potent strategy for brain cancer therapeutics [82]. The authors developed a natural and safe transportation system using focused ultrasound to increase the targeted delivery of doxorubicin-loaded exosomes for glioma therapy [82]. 

### 7.6. Thyroid Cancer

Thyroid cancer (TCa) usually has a good prognosis, but there is a subset of patients for whom standard care treatment is limited to surgery or surgery plus radioactive iodine is not sufficient. In case of aggressive thyroid tumors treatment include tyrosine kinase inhibitors, mitogen-activated protein kinase inhibitors, and mammalian target of rapamycin inhibitors [125]. Exosomal miR-485-3p and miR-4433a-5p in plasma might serve as biomarkers for papillary thyroid cancer (PTC) diagnosis, whereas miR-485-3p could also enable discrimination between high- and low-risk PTC [126].

Using a dioctadecyl-tetramethylindodicarbocyanine-4-chlorobenzenesulfonate salt label on exosomes extracted from CaL62/Rluc cells visualized the development of TCa in the body. This, in turn, provided a tissue and tumor edges imaging system for reducing surgical errors [127].

### 7.7. Ovarian Cancer

Ovarian cancer (OC) is the most lethal gynecological malignancies with a high capacity for metastasis [128]. The detection is based on markers like the cancer antigen 125 (CA-125), 19-9 (CA19-9), the epithelial cell adhesion molecule (EpCAM), CD24, the human epidermal growth factor receptor 2 (HER2), mucin 18 (MUC18), the epidermal growth factor receptor (EGFR) and claudin 3 (CLDN3) [129]. OC exosomes are key biomarkers to liquid biopsy and targets of chemotherapy [130,131]. 

In the study of Molavipordanjani et al., ^99^mTc-radiolabel HER2 targeted exosomes (^99^mTcexosomes) were provided for ovary tumor imaging [93]. These exomes were obtained from genetically engineered cells and possessed a ligand for HER2 receptors. The biodistribution study in SKOV-3 tumor-bearing nude mice confirmed the ability of ^99^mTc-exosomes to accumulate and visualize a tumor in a SKOV-3 tumor-bearing nude mouse [93].

### 7.8. Kidney Cancer

Malignant kidney tumors account for 2% of the global cancer burden with hard to detect and difficult to treat [132]. Clear cell renal cell carcinoma (ccRCC) is the most common renal cancer in adults. Differentially expressed genes (DEGs), including VEGFA, PPARA, CCND1, FLT1, CXCL12, FN1, DCN and ERBB4 were identified as biomarkers of ccRCC [133]. miR-204-5p in urinary exosomes could be a useful biomarker for early diagnosis of patients with renal cell carcinoma (Xp11 tRCC) [134]. 

Engineered exosomes from the human embryonic kidney 293T, Gaussia luciferase, and metabolic biotinylation for the creation of a sensitive EV reporter (EV-GlucB) enabled the multimodal in vivo imaging and EV level monitoring in ex vivo organs and biofluids [135]. 

### 7.9. Melanoma 

Cutaneous melanoma is the deadliest form of skin cancer. In high-risk patients, such as thick primary tumors, or following treatment of metastases, US of regional lymph node, CT, or whole-body PET/PET-CT scans may lead to earlier diagnosis. Immunotherapy and targeted therapy demonstrate favorable effects in patients with a low tumor burden. Rising levels of the serum S100 protein has a higher specificity for disease progression [136]. The cell-free microRNA (cf-miRNAs) panel, such as the serum of cf-miR-9-5p, cf-miR-145-5p, cf-miR-150-5p, cf-miR-155-5p, and cf-miR-205-5p is used to detect the presence of metastasis in patients with melanoma [137]. 

The surface functionalization of plasmonic gold nanoparticles was used by Lara et al. to advance the indirect labeling of EVs without affecting size distribution, polydispersity, surface charge, protein markers, cell uptake, or in vivo biodistribution [65]. Double-labeled EVs with gold and fluorescent dyes were infused into animals creating metastatic lung nodules and investigated by fluorescence/computer tomography imaging, quantitative neutron activation analysis, and gold-enhanced optical microscopy. The most noteworthy accumulation was seen in melanoma metastatic tumor tissue treated with gold-nanoparticles conjugated EV. This indicates that EVs not only reach but also take up tumors to deliver their gold nanoparticle-cargo [65]. 

Melanoma B16F10-derived exosomes (ExoB16) were radiolabeled with intraluminal labeling (entrapment of ^111^Indium via tropolone shuttling); and membrane labeling (chelation of ^111^Indium via covalently attached bifunctional chelator DTPA-anhydride) [94]. Membrane exosome radiolabeling enables accurate live imaging and quantitative biodistribution studies [94]. Exosome modification allows for exosome imaging and tracking in vivo for nanomedicine applications [4]. Melanoma exosomes can be imaged in vitro, and within lymph nodes in vivo with the use of standard MRI approaches by using a C57BL/6 mouse model [69]. 

### 7.10. Osteosarcoma

Osteosarcoma is the most commonplace malignant primary bone tumor in young adults and children. Osteosarcoma growth takes place in the most active sites, namely, the metaphysis regions of long bones [138]. 

MRI was applied in exosome accumulation within ectopic osteosarcoma tumor-bearing mice [91]. Gadolinium, an MRI contrast agent, was used to label exosomes derived from the human umbilical cord mesenchymal stromal cells. Results suggested that the human umbilical cord’s mesenchymal stromal cell exosomes labeled with gadolinium do actually accumulate within human or mouse osteosarcoma cells in vitro and in vivo after infusion [91]. Rayamajhi and colleagues analyzed macrophage-derived extracellular vesicles for their contrast enhancement ability in the tumor area in osteosarcoma tumor-bearing mice [77]. It was concluded that using macrophage cell-derived extracellular vesicles reconstructed with a Gd-conjugated liposomal system enables a three-fold reduction in clinically used gadolinium concentration while maintaining contrast in the clinical window. Besides, reconstructed EVs indicated a preferential cellular interaction and collection towards cancer cells compared to non-cancer cells in vivo [77].

## 8. Future Perspectives of the Exosomes in Imaging and Theranostic Application

In the following decade, EVs may rose as a key cell-free technique for the diagnosis of a scope of pathologies, including cancer. The field is quickly progressing from promising in vitro reports towards in vivo animal models and early pre-clinical investigations. The EVs-mediated delivery of contrast media and drugs is a promising tool for cancer imaging and theranostics. It may function as a radionuclide or dye and as a delivery vehicle of anticancer drugs. This could represent a critical advance forward to the disclosing of exosome homing and, more broadly, TEX in pathological conditions. The route of EV administration determines bioavailability and affects its therapeutic effect [62]. Despite advances in exosome modification strategies, there are still many challenges to overcome to effectively harness their therapeutic potential. Future research requires optimizing exosomal nanocarrier formulation, matching with nanomedicine applications, and investigating their interaction with the immune system. Progressing studies regarding the cargo contents of TEX are required and will assist with distinguishing important radiological contrast agents and targeting residues. This research facilitates personalized exosome-based nanomedicine [4]. Further studies on exosomes can be conducted to fully utilize their potential in translational medicine. This can serve to create effective clinical diagnostics and therapeutic strategies [15]. Exosomes applied in an individualized, targeted nanomedicine setting have high potential to turn into the most important theranostics for the determination, diagnosis, and therapy of cancer. Subsequently, this field offers an energizing chance to research new bioengineering innovations and advance towards exceptionally viable diagnostic and therapeutic applications. Exciting times are in front of us as EVs are making progress towards being effective delivery agents for cancer therapy. 

## 9. Conclusions

The creation of contrast media based on exosomes is an inventive strategy and opens up the chance of imaging in vivo with a forceful and noninvasive method, such as MRI, CT, PET, and hybrid imaging. Researchers exploited a lot of nanoparticles and agents for their utility in diagnostic imaging. Thus, the compatibility of exosomes with different contrast media provides an opportunity to develop novel approaches in cancer diagnosis. Exosomes can deliver anticancer therapeutics to cancer cells and simultaneously be imaged using radiological methods. This dual capacity of exosomes makes them unique for developing theranostics. Likewise, the improvement of exosome-based diagnostic strategies for early cancer detection and for treating disease denotes a new era of precision radiology and nuclear medicine in the 21st century. 

## Figures and Tables

**Table 1:** Modification strategies of exosomes for applications in contrast media and radionuclide composition as well as theranostic development.

Exosome Modification Technique	Advantages	Disadvantages
Direct incubation	The simplest approach for loading functional molecules into the cavities of exosomes	Low loading efficiency, lack of selectivity
Electroporation	Stimulation of external electric fields and production of nano-sized pores on the surface through which functional molecules can enter into the inner spaces	Loading of hydrophobic agents may be inefficient
Sonication	The shear force generates nano-sized pores on the surface of the membrane letting the molecules diffuse into the cavity	The recovery of the membrane after sonication took approximately one hour and is required to reduce the leakage of the packaged molecules
Phospholipid substitution	Dynamic exchange of the phospholipid between the cells and the phospholipid derivate within the culture medium	Phospholipid derivate could only target tumor cells expressing the folate receptor
Covalent coupling	Functionalisation of exosomes with antibodies and other functional molecules by chemical coupling	Surface constitution of exosomes is too complicated, and the surfaces lack relevant active functional groups which are necessary for covalent coupling
Aptamer technique	Direct and selective engineering of the surface of exosomes with functional DNA	Unstable delivery of these membrane molecules to the shedding EVs
Gene engineering	Giving exosomes different functions as they are directly shed from the membranes of the engineered maternal cells	Poor expression of these membrane molecules to the shedding EVs

**Table 2:** Data showing the potential role of EVs as a contrast media or radiolabel in radiology and nuclear medicine.

Imaging Modality	EVs Origin	Type of Contrast Media /Radiolabel	Application	Reference
Computed tomography	Melanoma cells	Folic acid-conjugated gold nanoparticles	Melanoma	Lara, P., J Nanobiotechnology, 2020 [65]
Computed tomography	mesenchymal stem cells	Glucose-coated gold nanoparticles	Brain	Perets, N., Nano Lett., 2019 [66]
Computed tomography	Mesenchymal stem cells	Glucose-coated gold nanoparticle	Brain	Betzer, O., ACS Nano, 2017 [67]
Magnetic resonance imaging	Macrophage cell	Gadolinium infused liposomes	Mouse osteosarcoma	Rayamajhi, S., Biomater Sci, 2020 [77]
Magnetic resonance imaging	Human umbilical cord mesenchymal stromal cells	Gadolinium	Osteosarcoma	Abello, J., Theranostics 2019 [91]
Magnetic resonance imaging	Mesenchymal stem cells	Ferritin heavy chain	Different conditions	Liu, T., Magn. Reson. Imaging, 2020 [76]
Magnetic resonance imaging	Human bone marrow mesenchymal stem cells	Superparamagnetic iron oxide nanoparticles conjugated with rhodamine	Phantom	Dabrowska, S., Int J Nanomedicine, 2018 [75]
Magnetic resonance imaging	Melanoma cells	Superparamagnetic iron oxide nanoparticles	Lymph nodes	Hu, L., Magn. Reson. Med., 2015 [69]
Magnetic resonance imaging	Melanoma cells	Superparamagnetic iron oxide nanoparticles	Phantom	Hood, J.L., Anal. Biochem., 2014 [68]
Magnetic resonance imaging	Mesenchymal stem cells	Ultrasmall superparamagnetic iron oxide nanoparticles	Animal model	Busato, A., Int J Nanomedicine, 2016 [70]
Single-photon emission computed tomography	Macrophage cell line	Technetium derivative	Whole body	Hwang, D.W., Sci. Rep., 2015 [86]
Single-photon emission computed tomography	Erythrocyte	^99^mTc-tricarbonyl complex	Whole body	Varga, Z., Cancer Biother. Radiopharm., 2016 [87]
Gamma counter	4T1, MCF-7, and PC3 cancer cell lines	Indium-oxine	Tumor-bearing mouse models	Smyth, T., J. Control. Release, 2015 [88]
Single-photon emission computed tomography	Natural milk	^99^mTcCl_4_	Biodistribution	Gonzalez, M.I., Nanomaterials (Basel), 2020 [89]
Positron emission tomography	Mouse liver proliferative cells	[^124^I]Na	Biodistribution	Royo, F., Nanoscale, 2019 [90]
Positron emission tomography	4T1 breast cancer cells	(^64^Cu)-radiolabeled polyethylene glycol	Tumor uptake	Shi, S., Bioconjug. Chem., 2019 [92]
Single-photon emission computed tomography	Genetically engineered cells	[^99^mTc(CO)3(H2O)3]	HER2 receptors	Molavipordanjani, S., Eur. J. Pharm. Sci., 2020 [93]
Single-photon emission computed tomography	Melanoma (B16F10) cells	^111^Indium	Whole body	Faruqu, F.N., Theranostics, 2019 [94]

**Table 3:** Studies on multimodal imaging of EVs.

Modalities	EVs Origin	Type of Contrast Media/Radiolabel	Application	Reference
Computed tomography Magnetic resonance imaging	HeLa, HepG2, and L02 cell lines	Iridium and iron oxide nanoclusters	Tumor-target	Shaikh, S., ACS Appl Mater Interfaces, 2018 [95]
Computed tomography Magnetic resonance imaging	HepG2 cell line	Silver and iron oxide nanoclusters	Cancer	Tayyaba, R.F.U., J Mater Chem B 2020, [96]
Magnetic resonance imaging Positron emission tomography	Human umbilical cord blood mononuclear cells	^64^CuCl_2_	Brain, liver	Banerjee, A., Nanoscale, 2019 [71]

**Table 4:** Therapeutic and diagnostic application of EVs in a treatment attempt.

Modality	EVs Origin	Type of Contrast Media	Therapeutic Mechanism/Application	Reference
Magnetic resonance imaging	Cancer cell lines (4T1, HepG2, and SKBR3)	Gold–iron oxide nanoparticles	Anti-miR-21. Phototherapy treatment.	Bose, R.J.C., ACS Nano, 2018 [97]
Magnetic resonance imaging	MDA-MB-231 human breast cancer cells	Superparamagnetic iron oxide nanoparticles	Olaparib (PARP inhibitor)	Jung, K.O., Biomaterials, 2018 [104]
Magnetic resonance imaging	Macrophage cells	Iron oxide nanoparticles	Chemotherapeutic agent (doxorubicin), tissue plasminogen activator (t-PA) and two photosensitizers	Silva, A.K., Nanomedicine, 2015 [101]
Magnetic resonance imaging	Lung fibroblast cell	Super paramagnetic iron oxide nanoparticle	Anticancer drug (doxorubicin)	Srivastava, A., Sci. Rep., 2016 [103]
Magnetic resonance imaging	Macrophage cell line Raw264.7	Superparamagnetic iron oxide nanoparticles	Curcumin (Cur) and neuropilin-1-targeted peptide	Jia, G., Biomaterials, 2018 [102]
